# The Alzheimer's Association Global Biomarker Standardization Consortium (GBSC) plasma phospho‐tau Round Robin study

**DOI:** 10.1002/alz.14508

**Published:** 2025-02-05

**Authors:** Nicholas J. Ashton, Ashvini Keshavan, Wagner S. Brum, Ulf Andreasson, Burak Arslan, Mathias Droescher, Stefan Barghorn, Jeroen Vanbrabant, Charlotte Lambrechts, Maxime Van Loo, Erik Stoops, Shweta Iyengar, HaYeun Ji, Xiaomei Xu, Alex Forrest‐Hay, Bingqing Zhang, Yuling Luo, Andreas Jeromin, Manu Vandijck, Nathalie Le Bastard, Hartmuth Kolb, Gallen Triana‐Baltzer, Divya Bali, Shorena Janelidze, Shieh‐Yueh Yang, Catherine Demos, Daniel Romero, George Sigal, Jacob Wohlstadter, Kishore Malyavantham, Meenakshi Khare, Alexander Jethwa, Laura Stoeckl, Johan Gobom, Przemysław R. Kac, Fernando Gonzalez‐Ortiz, Laia Montoliu‐Gaya, Oskar Hansson, Robert A. Rissman, Maria C. Carrillo, Leslie M. Shaw, Kaj Blennow, Jonathan M. Schott, Henrik Zetterberg

**Affiliations:** ^1^ Department of Psychiatry and Neurochemistry Institute of Neuroscience & Physiology the Sahlgrenska Academy at the University of Gothenburg Mölndal Sweden; ^2^ King's College London Institute of Psychiatry Psychology and Neuroscience Maurice Wohl Clinical Neuroscience Institute London UK; ^3^ NIHR Biomedical Research Centre for Mental Health and Biomedical Research Unit for Dementia at South London and Maudsley NHS Foundation Trust London UK; ^4^ Centre for Age‐Related Medicine Stavanger University Hospital Stavanger Norway; ^5^ Dementia Research Centre UCL Queen Square Institute of Neurology University College London London UK; ^6^ Graduate Program in Biological Sciences: Biochemistry Universidade Federal do Rio Grande do Sul (UFRGS) Porto Alegre Rio Grande do Sul Brazil; ^7^ Neuroscience Research AbbVie Deutschland GmbH & Co. KG Ludwigshafen Germany; ^8^ ADx NeuroSciences N.V. Ghent Belgium; ^9^ Alamar Biosciences, Inc. Fremont California USA; ^10^ ALZpath Inc. Carlsbad California USA; ^11^ Fujirebio Europe N.V. Ghent Belgium; ^12^ Enigma Biomedical Group San Diego California USA; ^13^ Neuroscience Biomarkers Janssen Research and Development San Diego California USA; ^14^ Clinical Memory Research Unit Department of Clinical Sciences Lund University Lund Sweden; ^15^ MagQu Co., Ltd. New Taipei City Taiwan; ^16^ Meso Scale Diagnostics, LLC. Rockville Maryland USA; ^17^ Quanterix Corp Billerica Massachusetts USA; ^18^ Roche Diagnostics GmbH Penzberg Germany; ^19^ Clinical Neurochemistry Laboratory Sahlgrenska University Hospital Mölndal Sweden; ^20^ Memory Clinic Skåne University Hospital Malmö Sweden; ^21^ Alzheimer's Therapeutic Research Institute Keck School of Medicine of the University of Southern California San Diego California USA; ^22^ Division of Medical & Scientific Relations Alzheimer's Association Chicago Illinois USA; ^23^ Department of Pathology & Laboratory Medicine Perelman School of Medicine University of Pennsylvania Philadelphia Pennsylvania USA; ^24^ Paris Brain Institute, ICM Pitié‐Salpêtrière Hospital Sorbonne University Paris France; ^25^ Neurodegenerative Disorder Research Center Division of Life Sciences and Medicine and Department of Neurology Institute on Aging and Brain Disorders University of Science and Technology of China and First Affiliated Hospital of USTC Hefei P.R. China; ^26^ UK Dementia Research Institute University College London London UK; ^27^ Department of Neurodegenerative Disease UCL Queen Square Institute of Neurology, Queen Square London UK; ^28^ Hong Kong Center for Neurodegenerative Diseases, Science Park Hong Kong China; ^29^ School of Medicine and Public Health University of Wisconsin‐Madison Madison Wisconsin USA

**Keywords:** Alzheimer's disease, candidate reference materials, cerebrospinal fluid, commutability, immunoassay, phosphorylated tau, plasma

## Abstract

**INTRODUCTION:**

The Alzheimer's Association Global Biomarker Standardization Consortium conducted a blinded case–control study to learn which phosphorylated tau (p‐tau) assays provide the largest fold‐changes in Alzheimer's disease (AD) versus non‐AD and show commutability in measuring patient samples and candidate certified reference materials (CRMs).

**METHODS:**

Thirty‐three different p‐tau assays measured paired plasma and cerebrospinal fluid (CSF) from 40 participants (25 with “AD pathology” and 15 with “non‐AD pathology” by CSF amyloid beta [Aβ]42/Aβ40 and p‐tau181 criteria). Four CRMs were assessed.

**RESULTS:**

Plasma p‐tau217 demonstrated higher fold‐changes between AD and non‐AD than other p‐tau epitopes. Fujirebio LUMIPULSE G, UGOT IPMS, and Lilly MSD p‐tau217 provided the highest fold‐changes. Plasma p‐tau217 showed the strongest correlations between plasma assays (rho = 0.81–0.97). The CRMs were not commutable across assays.

**DISCUSSION:**

Plasma p‐tau217 showed larger fold‐changes and better accuracy for detecting AD pathology in symptomatic individuals, with greater cross‐platform agreement than other p‐tau variants. Further work is needed to develop suitable CRMs facilitating cross‐assay standardization.

**Highlights:**

- Paired plasma and cerebrospinal fluid (CSF) samples from twenty‐five Alzheimer's disease (AD) and 15 non‐AD patients were measured blind.
- Thirty‐three plasma assays were compared, for phosphorylated tau‐181 (p‐tau181), 205, 212, 217 and 231.
- Plasma p‐tau217 consistently had the highest fold‐change and was best correlated between assays.
- Plasma‐CSF correlations were weak to moderate.
- There was lack of commutability for four candidate reference materials.

## 1 BACKGROUND

The neuropathological confirmation of amyloid β (Aβ) plaques and tau neurofibrillary tangles (NFT) remains the gold standard for a definitive diagnosis of Alzheimer's disease (AD). However, the clinical assessment of AD is being supported increasingly by validated positron emission tomography (PET) imaging and cerebrospinal fluid (CSF) biomarkers accurately reflecting Aβ “A”, tau “T”, and neurodegeneration “N” pathologies, which have improved the accuracy in diagnosing AD during life, and provided evidence for a biological classification of the disease (AT[N]).^1^ Yet, such biomarkers are considered to be specialized and have significant constraints (e.g., invasiveness and skill for CSF sampling, and cost for PET imaging), hindering their use as general tools for diagnosing and managing dementia in health systems across the globe.

Blood biomarkers capable of detecting core AD pathologies have demonstrated huge potential for clinical practice use, and in determining eligibility for, and response to, novel treatments. ^2^, ^3^ Plasma Aβ peptides (Aβ42/Aβ40),^4^, ^5^, ^6^ neurofilament light (NfL),^7^, ^8^ and glial fibrillary acidic protein (GFAP)^9^, ^10^ have all been shown to associate with certain AD features, but none can demonstrate the high disease specificity of plasma phosphorylated tau (p‐tau). Increased p‐tau is initially associated with Aβ deposition in asymptomatic individuals; further increases are seen in the symptomatic phases of AD, when overt NFT pathology is present in the brain and driving cognitive symptoms.

Tau is present and detectable in blood in various phosphorylated forms, including (but not limited to) p‐tau181, p‐tau205, p‐tau212, p‐tau217, and p‐tau231. Non‐p‐tau is also detectable in blood, either as “total‐tau,”^11^ “brain‐derived” tau,^12^ “N‐terminal tau (NTA)” tau,^13^ or as non‐phosphorylated peptide forms.^14^ Despite significant changes in symptomatic disease, non‐p‐tau species have limited utility in AD diagnostics but have possible applications in acute neurological conditions^15^ or more advanced disease stages.^13^ For the most part, p‐tau epitopes in blood exhibit a similar pattern of increase as AD pathology develops. However, distinctions have been reported between p‐tau forms in terms of diagnostic accuracy in symptomatic individuals,^16^, ^17^, ^18^ relationships with in vivo and post‐mortem pathology,^19^, ^20^ preclinical detection,^21^, ^22^ physiological fluctuations,^23^ and longitudinal change.^21^ These findings suggest that constructing disease staging based on biofluid measures of tau may be feasible.^24^

**RESEARCH IN CONTEXT**

1. **Systematic review**: We searched without language restrictions in PubMed for articles from January 1, 2014 to March 1, 2024, using the terms (((ℌAlzheimer Diseaseℍ[Mesh]) OR ((Alzheimer[Title/Abstract]) OR (Alzheimer's [Title/Abstract))) AND (((ℌBloodℍ[Mesh]) OR (ℌPlasmaℍ[Mesh])) OR (plasma[Title/Abstract]))) AND ((phospho tau[Title/Abstract]) OR (p‐tau[Title/Abstract]) OR (p‐tau[Title/Abstract]) OR (P‐tau[Title/Abstract]) OR (pTau[Title/Abstract])), filtering for studies in humans. Several studies examined plasma phosphorylated tau‐181 (p‐tau181), p‐tau217, and p‐tau231 in well‐characterized research cohorts including cognitively impaired and unimpaired individuals, demonstrating high diagnostic accuracy in relation to cerebrospinal fluid (CSF) or amyloid positron emission tomography (PET) imaging “gold standard” biomarkers, or post‐mortem amyloid and tau pathology. Most studies utilized single‐method assays. Some later studies have performed cross‐method and cross‐phospho‐form comparisons. No study to date has described comparisons with p‐tau212 and p‐tau205 in plasma, or assessed commutability of candidate reference materials (CRMs) between assays in comparison to patient samples.
2. **Interpretation**: Our study performed a blinded comparison of 33 plasma assays (for p‐tau181, p‐tau205, p‐tau212, p‐tau217, and p‐tau231). Among these were included several semi‐automated and fully automated methods that have potential for widespread clinical application. This is also the first study, to our knowledge, in which the commutability of CRMs was assessed for plasma p‐tau, as a first step in efforts to standardize between assays. We found that the top 10 assays in terms of fold‐change in plasma were p‐tau217 assays. Although plasma p‐tau217 measurements in patient samples across assays were highly correlated, we did not observe commutability of the four types of CRMs.
3. **Future directions**: Our study adds to the growing evidence for plasma p‐tau217 as a candidate biomarker for translation into clinical practice by virtue of its superior discriminative ability between AD and non‐AD in symptomatic individuals, when compared head‐to‐head with other p‐tau forms. The observed high and clinically interpretable fold‐changes are pivotal for this biomarker's possible future success. Further studies should examine whether blood biomarker–supported diagnosis will extend access to disease‐specific (and potentially disease‐modifying) treatments and impact patient‐relevant outcomes such as quality of life, particularly in diverse and resource‐limited settings. Standardization between different assays will also be important for real‐world applications, and this will require further concerted efforts in developing reference materials.

In addition to any possible pathophysiological differences between phospho‐forms, different quantification methods may also differentially influence results. Since the initial studies piloting p‐tau detection in blood,^25^, ^26^ several variations of antibody‐based technologies have been developed for quantification at femtomolar concentrations (e.g., Single‐Dimolecule array [Simoa],^27^, ^28^ immunomagnetic reduction [IMR],^29^ electrochemiluminescence [e.g., MesoScale Discovery,^30^ Elecsys, Roche Diagnostics International Ltd, Rotkreuz, Switzerland],^31^ and immunoprecipitation mass spectrometry [IPMS]^14^, ^32^). As recently approved anti‐Aβ therapies for AD approach clinical implementation, the use of numerous validated measures of blood p‐tau will likely guide timely treatment decisions. Several studies have already compared different p‐tau immunoassay platforms to detect a binary categorization of AD pathology.^16^, ^17^, ^33^, ^34^, ^35^ Yet it is also important to understand the translatability of different plasma p‐tau results across multiple platforms.

In this Alzheimer's Association Global Biomarker Standardization Consortium (GBSC) plasma p‐tau Round Robin study, we performed a comprehensive and blinded comparison of 33 different p‐tau assays, including seven different p‐tau epitopes, or p‐tau/t‐tau ratios, utilizing eight immunological platforms, in plasma and CSF from symptomatic individuals categorized as having AD or non‐AD pathology. Our main aim was to compare all assays regarding their ability to detect AD pathology (focusing on fold‐change between AD and non‐AD groups), correlations between plasma biomarkers and assays, and relationships with CSF p‐tau. A secondary aim was to test the commutability of four candidate certified reference materials (CRMs), that is, the consistency of relationships between assays in measuring the CRMs compared with the participant samples.

## 2 METHODS

### 2.1 Participants, ethics, and study design

Individual de‐identified ethylenediaminetetraacetic acid (EDTA) plasma and CSF samples were from the prospective Wolfson CSF study 12/0344 (PI Schott; NRES London Queen Square August 2013) at the University College London Dementia Research Centre. All individuals were being investigated by lumbar puncture for cognitive concerns after having been assessed in the specialist cognitive disorders service at the National Hospital for Neurology and Neurosurgery, University College London Hospitals NHS Trust, London, UK. Participants gave informed written consent to opportunistic research sample donation at the same time as sampling of their CSF and paired venous plasma for diagnostic purposes. Participant samples were collected serially over the period December 2020 to June 2022, and selected based on known CSF Aβ42/Aβ40 and spanning a range of p‐tau181 (LUMIPULSE G) concentrations measured previously in clinical routine, and availability of sufficient bio‐banked CSF (total 4 mL) and plasma (total 7 mL). These total volumes were determined after surveying all prospective participating labs to ascertain their minimal and ideal volumes of CSF and plasma required to carry out their respective assays. A participant was considered to have “AD pathology” if the CSF results were Aβ42/Aβ40 <0.065 and p‐tau181 >57 pg/mL.^36^ Plasma and CSF aliquots of 1 mL were sent on dry ice to the University of Gothenburg for sub‐aliquoting and distribution to participating laboratories/assay developers, who were blinded to participant information.

### 2.2 CSF and plasma collection

Participants were not instructed to fast, and CSF sampling was performed between 0800 and 1200 hours. After local anesthesia with lignocaine, a 22‐gauge atraumatic spinal needle was used to collect up to 20 mL of CSF, without active withdrawal, into 2 × 10 mL polypropylene screw top containers (Sarstedt 62.610.018), which were transported at ambient temperature within 30 min to the laboratory. CSF was centrifuged at 1750 *g* for 5 min at 4°C and the supernatant placed in 1 mL aliquots into polypropylene screw top cryovials. Peripheral venous blood was sampled using a tourniquet and 21‐gauge or 23‐gauge butterfly needle with a BD Vacutainer collecting system, into 6 mL K3‐EDTA plasma tubes, which were transported and centrifuged at ambient temperature, at 1800 *g* for 5 min, within 30 min of sampling. Plasma supernatant aliquots of 1 mL were stored in polypropylene screw‐top cryovials. Both CSF and plasma were stored at −80°C within 60 min of sampling.

### 2.3 p‐tau assays

Eleven participating centers received plasma and CSF aliquots. In total, 31 single p‐tau measurements (11 p‐tau181, 1 p‐tau205, 1 p‐tau212, 13 p‐tau217, and 5 p‐tau231) across eight immunological platforms were compared. In addition, we also included two p‐tau/tau ratios derived from mass spectrometric measurements: p‐tau205/tau205 (i.e., p‐tau205/tau195‐209) and p‐tau217/tau217 (i.e., p‐tau217/tau212‐221). All measurements were made in duplicate, except for those undertaken on the fully automated instruments (LUMIPULSE G, Fujrebio Europe N.V., Ghent, Belgium, and Cobas e 801 analyzers, Roche Diagnostics International Ltd, Rotkreuz, Switzerland) and NULISA. Each assay was performed in plasma and CSF except for the Elecsys pTau217 prototype immunoassay (Elecsys p‐tau217; Roche Diagnostics International Ltd, Rotkreuz, Switzerland) and the UGOT IPMS, which were not available for CSF. An overview of the immunological platforms is shown in Table 1 (Table S1, Appendix p. 17, if assay procedures differ for CSF**)**. Methods have been described previously for ADx Simoa p‐tau181,^33^ ALZpath p‐tau217,^37^ Janssen Simoa p‐tau217,^28^, ^38^ Fujirebio Lumipulse G pTau181 (Plasma),^17^ MSD Lilly p‐tau181 and p‐tau217,^30^, ^39^ MagQu p‐tau181,^29^ Meso Scale S‐PLEX p‐tau181^40^ and p‐tau217^41^, Quanterix Simoa p‐tau181 v2.1,^33^ Roche Elecsys p‐tau181 (Roche Diagnostics International Ltd, Rotkreuz, Switzerland),^31^ UGOT p‐tau181,^27^, ^42^ UGOT p‐tau212,^43^ UGOT p‐tau217,^44^ UGOT p‐tau231,^45^, ^46^ and UGOT IPMS.^14^ Method descriptions for Abbvie Erenna p‐tau217 and p‐tau231, ADx Lumipulse G p‐tau217, ADx Simoa p‐tau217, Alamar Biosciences NULISA p‐tau181, p‐tau217 and p‐tau231, Fujirebio Lumipulse G pTau217 Plasma RUO, Meso Scale S‐PLEX p‐tau231, and Roche Elecsys p‐tau217 are detailed in the Supplementary Methods (Appendix p. 3,4). All assay measures were performed by assay vendors. CSF and plasma ALZpath p‐tau217 were measured at the Department of Neurochemistry, University of Gothenburg. CSF and plasma MSD Lilly p‐tau217 and p‐tau181 were measured at the Clinical Memory Research Unit, Lund University. The analytical performance of the assays in terms of repeatability, intermediate precision, and sample performance is shown in Table S2 (Appendix p. 20).

**TABLE 1:** Plasma assay characteristics.

Participating center	Target	Analytical platform	Functional LLOQ	LOD	Sample volume for duplicate (dead volume)	Sample dilution (sample diluent)	Calibrator	Calibrator range	Capture antibody	Detector antibody	Other assay details/Reference
Abbvie	p‐tau217	Erenna	0.15 pg/mL	0.05 pg/mL	20 µL (3 µL)	x10 (SMC Standard Diluent, Merck)	Full‐length tau 441 expressed & phosphorylated in vivo by Sf9 cells	0–36.45 pg/mL	ab288167 (epitope phosphorylated at T217)	Tau12 (N‐terminal aa 6‐18)	Method in supplement
Abbvie	p‐tau231	Erenna	0.15 pg/mL	0.05 pg/mL	20 µL (3 µL)	x10 (SMC Standard Diluent, Merck)	Full‐length tau 441 expressed & phosphorylated in vivo by Sf9 cells	0–36.45 pg/mL	ab156624 (epitope phosphorylated at T231)	Tau12 (N‐terminal aa 6‐18)	Method in supplement
ADx NeuroSciences	p‐tau181	Simoa HD‐X	3.9 pg/mL	0.7 pg/mL	200 µL prediluted sample (30 µL)	x5 (Homebrew Sample Diluent, ADx)	Synthetic peptide covering epitopes of used antibodies	0–50 pg/mL	ADx252 (epitope phosphorylated at T181)	ADx204 (N‐terminal aa 6‐18)	2‐step HD‐X set‐up (80‐14c). 50% helper beads Bayoumy et al. 2021^33^
ADx NeuroSciences	p‐tau217	Simoa HD‐X	0.036 pg/Ml	0.008 pg/mL	200 µL prediluted sample (30 µL)	x3 (Homebrew Sample Diluent, ADx)	Synthetic peptide covering epitopes of used antibodies	0–50 pg/mL	RD‐84 (epitope phosphorylated at T217)	ADx204 (N‐terminal aa 6‐18)	2‐step HD‐X set‐up (80‐7c). 50% helper beads Method in supplement
ADx NeuroSciences	p‐tau217	LUMIPULSE G	0.020 pg/mL	0.008 pg/mL	200 µL neat sample (100 µL)	Neat (with addition of 20% Assay Specific Diluent—ASD)	Synthetic peptide covering epitopes of used antibodies	0–25.6 pg/mL	RD‐85 (epitope phosphorylated at T217)	ADx204 (N‐terminal aa 6‐18)	Specific 2‐step Lumipulse G set‐up (10‐10‐5 min.) Method in supplement
Alamar Biosciences, Inc	p‐tau181	NULISA qPCR (Singleplex) NULISAseq (Multiplex)	0.25 pg/mL	0.015 pg/mL	71 µL (31 µL)	Neat sample with x5 onboard	Full‐length recombinant tau 441 with site‐specific phosphorylation at T181	0–920 pg/mL	Proprietary	Proprietary	Fully automated, NUcleic acid Linked Immuno‐Sandwich Assay Method in supplement
Alamar Biosciences, Inc	p‐tau217	NULISA qPCR (Singleplex) NULISAseq (Multiplex)	0.25 pg/mL	0.019 pg/mL	71 µL (31 µL)	Neat sample with x5 onboard	Full‐length recombinant tau 441 with site‐specific phosphorylation at T217	0–920 pg/mL	Proprietary	Proprietary	Fully automated, NUcleic acid Linked Immuno‐Sandwich Assay Method in supplement
Alamar Biosciences	p‐tau231	NULISA qPCR (Singleplex) NULISAseq (Multiplex)	0.25 pg/mL (Singleplex)	0.015 pg/mL (Singleplex)	71 µL (31 µL)	Neat sample with x5 (singleplex) or x10 (multiplex) onboard	Full‐length recombinant tau 441 with site‐specific phosphorylation at T231	0–920 pg/mL	Proprietary	Proprietary	Fully automated, NUcleic acid Linked Immuno‐Sandwich Assay Method in supplement
ALZpath, Inc	p‐tau217	Simoa HD‐X	0.060 pg/mL	0.0074 pg/mL	100 µL (30 µL)	x3 for EDTA plasma and serum x10 to x30 for CSF	Synthetic peptide	0.012–50 pg/mL	ALZpath proprietary Ab (Rabbit monoclonal IgG epitope)	Mouse monoclonal IgG N‐terminus	2‐step HD‐X setup (35‐5). 75% helper beads Ashton et al. 2023^16^
Janssen R&D	p‐tau217	Simoa HD‐X	0.036 pg/mL	0.002 pg/mL	172 µL (30 µL)	x2 (custom)	Synthetic peptide (4.5 kDa) = epitope of capture Ab‐PEG4‐epitope of detection Ab	0–10 pg/mL	pT3 (epitope = 210‐220, phosphorylated at T217, with enhanced binding with phosphorylation at T212)	hT43 (N‐terminal aa 7‐20)	3‐step HD‐X setup (35‐5‐5). 75% helper beads. 25 µL RGP Triana‐Baltzer et al. 2021^28^
Fujirebio	p‐tau181	LUMIPULSE G	0.275 pg/mL	0.023 pg/mL	230 µL (100 µL)	Neat	pTau 181 synthetic peptide (75aa)	0–60 pg/mL	AT270 (epitope 176‐ 182 phosphorylated at T181)	BT2 (epitope 194‐198) and HT7 (epitope 159‐163)	2‐step Janelidze et al. 2023^17^
Fujirebio	p‐tau217	LUMIPULSE G	0.035 pg/mL	0.018 pg/mL	200 µL (100 µL)	Neat (with addition of 20% v/v Assay Specific Solution—ASS)	pTau 217 synthetic peptide	0–10 pg/mL	RD‐85 (epitope phosphorylated at T217)	BT2 (epitope 194‐198) and HT7 (epitope 159‐163)	Specific 2‐step Lumipulse G set‐up (10‐10‐5 min) Method in supplement
Lund University	p‐tau217	MSD Lilly	0.18 pg/mL	0.12 pg/mL**^b^**	60 µL (15 µL)	1:2 (Low salt buffer)**^c^**	Synthetic p‐tau217 peptide	0–100 pg/mL	Biotinylated‐IBA493 (anti‐p‐tau217)	SULFO‐TAG‐4G10‐E2 (Anti‐tau)	Palmqvist et al. 2020^38^
Lund University	p‐tau181	MSD Lilly	0.59 pg/mL	0.46 pg/mL**^b^**	60 µL (15 µL)	1:2 (Low salt buffer)**^c^**	Synthetic p‐tau181 peptide	0–100 pg/mL	Biotinylated‐IBA406 (anti‐p‐tau181)	SULFO‐TAG‐4G10‐E2 (Anti‐tau)	Janelidze et al. 2020^30^
MagQu	p‐tau181	IMR	0.0196 pg/mL	0.0196 pg/mL	120 µL (15 µL)	Neat	Synthetic p‐Tau 181peptide	0.0196–100 pg/mL	N/A	Phospho‐Tau (Thr181) Monoclonal Antibody	Yang et al. 2018^29^
Meso Scale Diagnostics, LLC. (MSD)	p‐tau181	MSD S‐PLEX	0.46 pg/mL	0.078 pg/mL	50 µL (10 µL)	Neat	Recombinant p‐tau expressed in a human cell line and confirmed by mass spectrometry to display phosphorylation at T181	0–2110 pg/mL	Human Tau (pT181) MSD Generation A Antibody Pair	Tau (total) MSD Generation A	Electrochemiluminescence (ECL) S‐PLEX assay Kivisäkk et al. 2023^40^
Meso Scale Diagnostics, LLC. (MSD)	p‐tau217	MSD S‐PLEX	1.81 pg/mL	0.29 pg/mL	50 µL (10 µL)	Neat	Recombinant p‐tau expressed in a human cell line and confirmed by mass spectrometry to display phosphorylation at T217	0–3880 pg/mL	Human Tau (pT217) MSD Generation A Antibody Pair	Tau (total) MSD Generation A	Electrochemiluminescence (ECL) S‐PLEX assay Kivisäkk et al. 2024^41^
Meso Scale Diagnostics, LLC. (MSD)	p‐tau231	MSD S‐PLEX	15 pg/mL	0.94 pg/mL	50 µL (10 µL)	Neat	Recombinant p‐tau expressed in a human cell line and confirmed by mass spectrometry to display phosphorylation at T231	0–40000 pg/mL	Human Tau (pT231) MSD Generation A Antibody Pair	Tau (total) MSD Generation A	Electrochemiluminescence (ECL) S‐PLEX assay Method in supplement
Quanterix Simoa	p‐tau181	Simoa HD‐X	8 pg/mL	0.62 pg/mL	80 µL (30 µL)	x4 (Sample Diluent)	Antigen in buffer with protein stabilizers	0–404 pg/mL**	Proprietary	Proprietary	2‐step HD‐X set‐up (35‐5). Bayoumy et al. 2021^33^
Roche Diagnostics International Ltd	p‐tau181	Cobas e (Elecsys)	0.300 pg/mL	≤0.300 pg/mL	30 µL (approx. 100 µL) singlicate	No dilution required	Proprietary	0.300–10.0 pg/mL	Proprietary	Proprietary	Electrochemiluminescence sandwich immunoassay, 1 min total incubation time Palmqvist et al. 2023^31^
Roche Diagnostics International Ltd	p‐tau217	Cobas e (Elecsys)	0.075 pg/mL	≤0.075 pg/mL	60 µL (approx. 100 µL) singlicate	No dilution required	Proprietary	0.0750–5.00 pg/mL	Proprietary	Proprietary	Electrochemiluminescence sandwich immunoassay, 18 min total incubation time Method in supplement
University of Gothenburg (UGOT)	p‐tau181	Simoa HD‐X	1 pg/mL	0.25 pg/mL	100 µL (30 µL)	x2 (Advantage diluent, Quanterix)	Full‐length recombinant tau 441 phosphorylated in vitro by GSK3β	0–128 pg/mL	AT270 (epitope 176‐ 182 phosphorylated at T181)	Tau12 (N‐terminal aa 6‐18)	3‐step HD‐X set‐up (40‐7‐7). 0% helper beads. Karikari et al. 2020^27^
University of Gothenburg (UGOT)	p‐tau212	Simoa HD‐X	0.073 pg/mL	0.01 pg/mL	240 µL (40 µL)	x1.2 (Tau 2.0, Quanterix)	Full‐length recombinant tau 441 phosphorylated in vitro by DYRK1A (Abcam269022)	0–41.67 pg/mL	p‐Tau212.7B3 (epitope p‐Tau212)	Tau12 (N‐terminal aa 6‐18)	2‐step HD‐X set‐up (47‐7). 0% helper beads. Kac et al. 2024^35^
University of Gothenburg (UGOT)	p‐tau217	Simoa HD‐X	0.4 pg/mL	0.08 pg/mL	150 µL (30 µL)	x1.5 (Tau2.0 diluent, Quanterix)	Full‐length recombinant tau 441 phosphorylated in vitro by GSK3β	0–53.7 pg/mL	Bioventix p.Tau217.FG (epitope phosphorylated at T217)	Tau12 (N‐terminal aa 6‐18)	2‐step HD‐X set‐up (47‐7). 0% helper beads. Gonzalez‐Ortiz et al. 2023^44^
University of Gothenburg (UGOT)	p‐tau231	Simoa HD‐X	1 pg/mL	0.25 pg/mL	100 µL (30 µL)	x2 (Advantage diluent, Quanterix)	Full‐length recombinant tau 441 phosphorylated in vitro by GSK3β	0–128 pg/mL	ADx253 (epitope 224‐240 phosphorylated at T231)	Tau13 (N‐terminal aa 2‐18)	3‐step HD‐X set‐up (40‐7‐7). 0% helper beads. Ashton et al. 2021^45^
University of Gothenburg (UGOT)	p‐tau181, p‐tau205, p‐tau217, p‐tau231, tau195‐209 tau212‐221	Mass Spectrometry	unknown	unknown	1000 µL (0 µL)	No dilution	Heavy labelled peptides	0.1–1 fmol	Tau12 (aa 6‐18), HT7 (aa 159‐163), BT2 (aa 194‐198)	n.a	UGOT Plasma Tau IP‐MS Imunnoprecipitation followed by LC‐MS. Targeted PRM MS method. Montoliu‐Gaya et al. 2023^14^

Abbreviations: aa, amino acid; Ab, antibody; DYRK1A, dual‐specificiyt tyrosine phosphorylation‐regulated kinase 1A; GSK3β, glycogen synthase kinase 3 beta; IP‐MS, immunoprecipitation‐mass spectrometry; LC‐MS, liquid chromatography‐mass spectrometry; LLOQ, lower limit of quantification; LOD, limit of detection; n.a., not applicable; p‐tau181, phosphorylated tau at threonine 181; p‐tau205, phosphorylated tau at serine 205; p‐tau217, phosphorylated tau at threonine 217; p‐tau231, phosphorylated tau at threonine 231

### 2.4 Candidate certified reference materials (CRMs)

Each plasma assay also assessed candidate CRMs created for this project. Briefly, 12 candidate CRMs (four CRMs [A–D] each at three different concentrations), were assessed (Table S3, Appendix p. 23). Candidate CRMs were either full‐length recombinant tau1–441 phosphorylated in vitro by glycogen synthase kinase 3β (TO8‐50FN; SignalChem, Vancouver, BC, Canada) in two buffers: Tau 2.0 Sample Diluent (Quanterix, #103847; A), phosphate‐buffered saline [PBS] + 0.05% Tween (B), or human EDTA plasma pool spiked with either full‐length recombinant tau tau1–441 phosphorylated in vitro by glycogen synthase kinase 3β (C) or human CSF (D). The human plasma pool used for CRM C and D was made from remnant de‐identified samples from individuals who had given plasma for clinical testing at Sahlgrenska University Hospital, who had not been diagnosed with AD as informed by clinical CSF testing. The human CSF used for CRM D was from remnant de‐identified samples from patients undergoing CSF testing for suspected AD. Concentrations of each candidate CRM were determined by the UGOT Simoa p‐tau181 assay,^27^ in duplicate across three independent runs. Each analytical laboratory in the study was instructed to measure the candidate CRM in duplicate and to treat them as unknown plasma samples.[Table alz14508-tbl-0002]

### 2.5 Statistical analysis

Demographic information was summarized using descriptive statistics. To evaluate the magnitude of biomarker increases in the AD versus non‐AD groups, mean and median fold‐changes were computed for each plasma and CSF biomarker assays, with forest plots showing the associated standard errors. The discriminative ability of a given plasma or CSF biomarker to detect confirmed AD pathology (using an AD CSF profile as the reference standard) was evaluated by computing the area under the receiver‐ operating characteristic (ROC) curve (AUC) and visualized with forest plots alongside 95% confidence intervals (CIs). To evaluate the associations between different assays for a given p‐tau biomarker (e.g., correlations between different p‐tau217 assays), we generated scatterplots alongside the between‐assay Spearman correlation coefficient and the Passing‐Bablok equation. For assays with available results in both plasma and CSF, we evaluated the cross‐matrix associations with Spearman correlation, calculated both in all patients and in the AD group. A two‐sided alpha of 0.05 was considered statistically significant. No multiple comparison adjustments were made, and the findings were interpreted accordingly. No CSF quantification of any participant sample fell below the limit of detection (LOD) for any assay. In plasma, measurements below the LOD were observed only for the Lilly p‐tau217 assay (*n* = 7). They were handled as described previously,^47^ by setting them to the LOD for this assay. In line with previous work with this assay, all (*n* = 7; 100%) of the observations occurred within the non‐AD CSF profile group. Where values returned as unable to be quantified, these individual sample results were omitted from the correlation analyses involving that assay alone. For candidate CRMs for plasma p‐tau217, the same approach of setting observations eventually falling below the LOD to the LOD value was followed. Several p‐tau217 assays presented values below the LOD for candidate CRMs as follows: ADx Lumipulse (A: *n* = 1, B: *n* = 2), ADx Simoa (B: *n* = 2), Janssen Simoa (A: *n* = 1, B: *n* = 4), Lilly MSD (A: *n* = 1, B: *n* = 2), MSD S‐Plex (B: *n* = 2), and Roche Elecsys (A: *n* = 1, B: *n* = 2, C: *n* = 1, D: *n* = 1).The 95% prediction interval (PI) of the Passing–Bablok regression was calculated to conclude whether the assessed CRM were commutable with the clinical individual samples based on the positions of their values with respect to the PI. All statistical analyses were performed in R v.4.2.1 (www.r‐project.org).

## 3 RESULTS

### 3.1 Participant characteristics

Of 75 possible participant samples, 40 were selected as having enough CSF and plasma volume available, with a wide range of clinical routine CSF p‐tau181 concentrations (20–295 pg/mL). Among these 40 participants (mean [SD] age, 63.8 [5.9] years; *n* [%] 17 female [42.5%]) (Table 2), 25 were categorized as having AD pathology and 15 as non‐AD pathology.

**TABLE 2 alz14508-tbl-0002:** Participant characteristics.

	All (*n* = 40)	CSF AD pathology (*n* = 25)	CSF non‐AD pathology (*n* = 15)
Mean age (SD), years	63.8 (5.8)	64.6 (6.1)	63.8 (5.4)
Female, *n* (%)	17 (42.5)	12 (48.0)	5 (33.3)
Ethnicity, *n* (%)	White 12 (30) Other 4 (10) Not available 24 (40)	White 9 (36) Other 2 (8) Not available 14 (56)	White 3 (20) Other 2 (13.3) Not available 10 (66.7)
Mean symptom duration (SD), months	53.2 (30.4)	44.0 (22.4)	68.6 (36.3)
Median CSF Aβ42 (IQR), pg/mL	315 (229, 433)	246 (224, 316)	507 (392, 613)
Median CSF Aβ40, (IQR), pg/mL	6583 (5189, 7752)	6584 (5292, 8906)	6581 (5107, 7403)
Median CSF Aβ42/40 (IQR)	0.045 (0.037, 0.079)	0.035 (0.038, 0.045)	0.083 (0.078, 0.088)
Median p‐tau181 (IQR), pg/mL	103 (41, 137)	124 (104, 165)	38 (29, 42)
Most recent clinical diagnosis	–	Amnestic AD Dementia (*n* = 20), mild cognitive impairment (*n* = 1), posterior cortical atrophy (*n* = 2), primary progressive aphasia (*n* = 2)	Subjective cognitive decline (*n* = 5), frontotemporal dementia not otherwise specified (*n* = 2), semantic variant primary progressive aphasia (*n* = 2), non‐fluent variant primary progressive aphasia (*n* = 1), meningioma and epilepsy (*n* = 1), alcohol‐related cognitive impairment (*n* = 1), Lewy body disease (*n* = 1), functional cognitive syndrome (*n* = 1), autoimmune encephalitis (*n* = 1)

*Note*: CSF biomarker values used for participant selection were obtained using the LUMIPULSE *G*1200 platform in clinical routine testing.

Abbreviations: Aβ40, amyloid‐beta 40; Aβ42, amyloid‐beta 42; AD, Alzheimer's disease; CSF, cerebrospinal fluid; IQR, interquartile range; p‐tau181, phosphorylated tau at threonine 181; SD, standard deviation.

### 3.2 Group‐wise differences of plasma and CSF p‐tau assays

**FIGURE 1:**
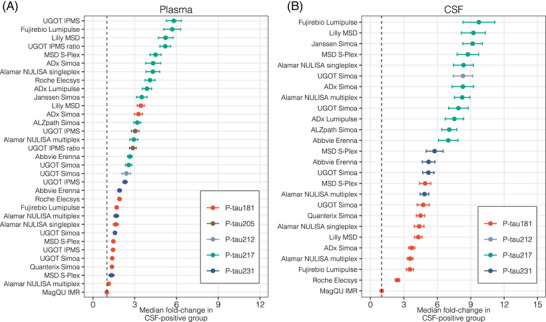
Median fold‐change of plasma and CSF p‐tau biomarkers in the AD versus non‐AD group. Forest plots indicate the median fold‐change of plasma (A) and CSF (B) p‐tau variants in the AD pathology group compared with the non‐AD pathology group. Bars correspond to standard error. Table S4 and Table S5 numerically describe this plot. AD, Alzheimer's disease; CSF, cerebrospinal fluid; IMR, immunomagnetic reduction; IPMS, immunoprecipitation‐mass spectrometry; MSD, MesoScale Discovery; NULISA, NUcleic acid Linked Immunosorbent Assay; p‐tau, phosphorylated tau; Simoa, single‐molecule arrray; UGOT, University of Gothenburg.

All CSF assays returned results above their respective lower limits of quantification (LLOQ) for all participant samples. In the case of plasma assays, the following assays had missing results due to values below the LLOQ (number of samples): ADx Simoa p‐tau181 (1), ADx Simoa p‐tau217 (5), Janssen Simoa p‐tau217 (3), ALZpath Simoa p‐tau217 (1), UGOT Simoa p‐tau217 (1), and UGOT Simoa p‐tau212 (1). Figure 1 shows the median fold‐change of p‐tau biomarkers in participants with AD pathology compared to those without AD pathology. For plasma (Figure 1A), the largest median fold‐changes were observed for assays targeting p‐tau217. UGOT IPMS (median fold‐change [standard error], 5.80 [2.70]), Fujirebio Lumipulse G (5.69 [3.05]), and Lilly MSD (5.49 [2.81]) all had median fold‐increases >5, whereas all other p‐tau217 assays had median fold‐increases ranging between 2.56 and 4.56 (Table S4, Appendix p. 24). In general, assays targeting other p‐tau epitopes had median fold‐changes <3 (Table S5, Appendix p. 25), with the exceptions being Lilly MSD p‐tau181 (3.43 [1.27]), ADx Simoa p‐tau181 (3.26 [1.47]), and UGOT IPMS p‐tau205 (3.04 [1.34]). Plasma p‐tau ratios (p‐tau217/tau217 and p‐tau205/tau205) did not increase the fold‐changes of the p‐tau assays alone. The individual boxplots for each plasma biomarker assay are shown in Figure S1 (p‐tau217), Figure S2 (p‐tau181), and Figure S3 (p‐tau231, p‐tau212, and p‐tau205) (Appendix p. 5–7. In CSF (Figure 1B), the highest median fold‐changes were demonstrated by assays targeting p‐tau217 (fold‐change range 6.98–9.76) but also p‐tau212 (8.31 [4.35]) compared to p‐tau231 (median fold‐change range 4.85–5.76) and p‐tau181 (fold‐change range 2.43–4.91, excluding MagQu). In CSF, the median fold‐changes were larger than in plasma for all assays. Nevertheless, the difference between plasma and CSF was more pronounced for p‐tau181, p‐tau212, and p‐tau231, whereas median fold‐changes for plasma and CSF p‐tau217 were more comparable. The Supplement displays results by mean fold‐change (Figure S4, Appendix p. 8; Tables S6 and S7; Appendix p. 26,27) and AUC ROC analysis (Figure S5, Appendix p. 9; Tables S8 and S9, Appendix p. 28,29**)**.

### 3.3 Correlations between plasma assays

**FIGURE 2:**
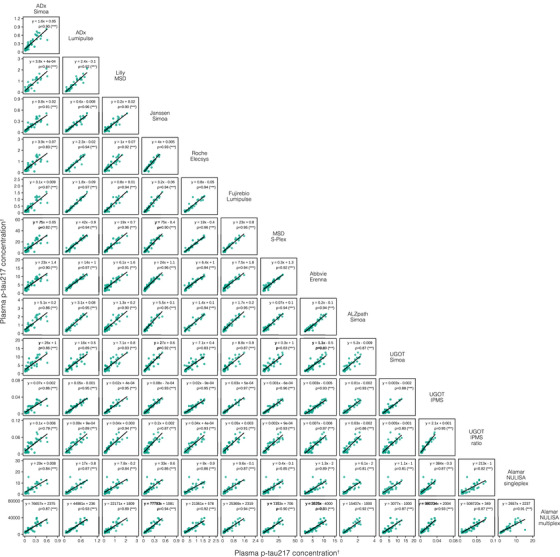
Correlations between all plasma p‐tau217 assays. Scatterplots represent the continuous associations between all plasma p‐tau217 assays. The dots indicate biomarker concentration and the solid black line indicates the mean regression line. In each panel, the text indicates the computed Passing–Bablok equation for each assay pair and Spearman's rho (*ρ*) alongside its level of statistical significance in brackets. ^†^Units are pg/mL for all assays excepting NULISA, which provides relative quantification in NPQ (NULISA Protein Quantification) units; IPMS, which is measured in fmol/L; and IPMS ratio, which has no units. ns, not significant. *p < 0.05, **p < 0.01, ***p < 0.0001. IPMS, immunoprecipitation‐mass spectrometry; MSD, MesoScale Discovery; NULISA, NUcleic acid Linked Immunosorbent Assay; p‐tau217, tau phosphorylated at threonine 217; Simoa, single‐molecule array; UGOT, University of Gothenburg.

**FIGURE 3:**
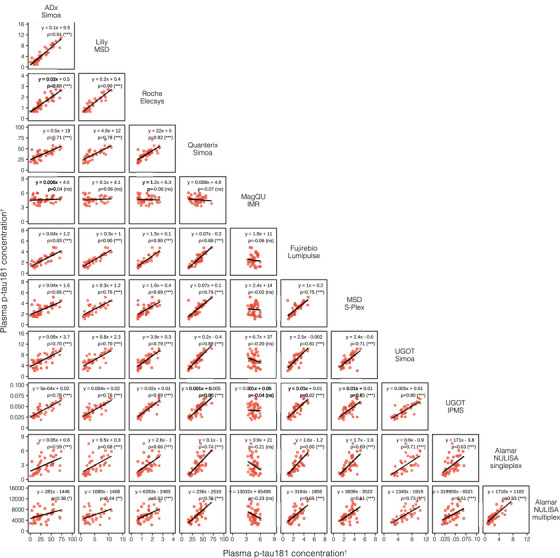
Correlations between all plasma p‐tau181 assays. Scatterplots represent the continuous associations between all plasma p‐tau181 assays. The dots indicate biomarker concentration and the solid black line indicates the mean regression line. In each panel, the text indicates the computed Passing–Bablok equation for each assay pair and Spearman's rho (*ρ*) alongside its associated level of statistical significance in brackets. ^†^Units are pg/mL for all assays except NULISA, which provides relative quantification in NPQ (NULISA Protein Quantification) units; IPMS, which is measured in fmol/L; and IPMS ratio, which has no units. ns, not significant. *p < 0.05, **p < 0.01, ***p < 0.0001. IMR, immunomagnetic reduction; IPMS, immunoprecipitation‐mass spectrometry; MSD, MesoScale Discovery; NULISA, NUcleic acid Linked Immunosorbent Assay; p‐tau181, tau phosphorylated at threonine 181; Simoa, single‐molecule array; UGOT, University of Gothenburg.

**FIGURE 4:**
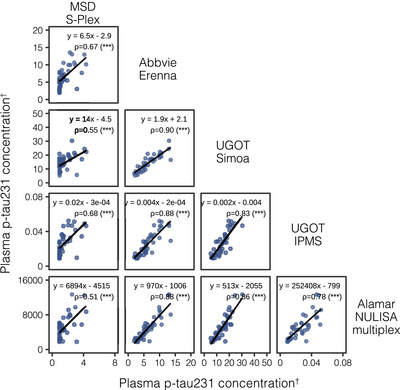
Correlations between all plasma p‐tau231 assays. Scatterplots represent the continuous associations between all plasma p‐tau231 assays. The dots indicate biomarker concentration and the solid black line indicates the mean regression line. In each panel, the text indicates the computed Passing–Bablok equation for each assay pair and Spearman's rho (*ρ*) alongside its level of statistical significance in brackets. ^†^Units are pg/mL for all assays except NULISA, which provides relative quantification in NPQ (NULISA Protein Quantification) units; IPMS, which is measured in fmol/L; and IPMS ratio, which has no units. ns, not significant. *p < 0.05, **, *p* < 0.01, ***p < 0.0001. IPMS, immunoprecipitation‐mass spectrometry; MSD, MesoScale Discovery; NULISA, Nucleic acid LInked Immunosorbent Assay; p‐tau231, tau phosphorylated at threonine 231; Simoa, single‐molecule array; UGOT, University of Gothenburg.

We examined the correlations between blood p‐tau biomarker assays, grouped by phosphorylation site (Figures 2, 3, 4). A stronger overall linear relationship was observed between p‐tau217 assays (mean rho = 0.90; rho range 0.79–0.97; Figure 2), compared to p‐tau181 (mean rho = 0.74; rho range 0.38–0.91, excluding MagQu; Figure 3) and p‐tau231 (mean rho = 0.75; rho range 0.51–0.89; Figure 4).

### 3.4 Correlation between plasma and CSF

**FIGURE 5:**
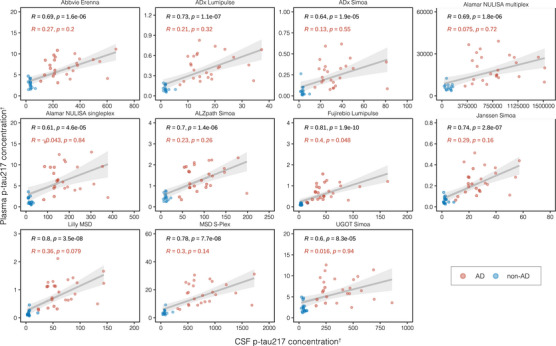
Intra‐assay correlations between plasma and CSF p‐tau217 biomarkers. Scatterplots represent the associations between biomarker measurements performed with the same assay in plasma (*y*‐axis) and CSF (*x*‐axis), alongside the mean regression line with 95% confidence intervals, computed based on data from all the participants in the cohort. Red dots indicate participants from the AD group and blue dots indicate participants from the non‐AD group, as defined per clinical evaluation and CSF Aβ42/Aβ40 status. In each panel, the text in black indicates the Spearman's correlation coefficient for the entire cohort and associated *p*‐value, with red text indicating the Spearman's correlation coefficient and associated *p*‐value for the AD group only. ^†^Units are pg/mL for all assays except NULISA, which provides relative quantification in NPQ (NULISA Protein Quantification) units. AD, Alzheimer's disease; CSF, cerebrospinal fluid; MSD, MesoScale Discovery; NULISA, NUcleic acid Linked Immunosorbent Assay, p‐tau217, tau phosphorylated at threonine 217; Simoa, single‐molecule array; UGOT, University of Gothenburg.

Next, we examined the correlation between plasma and CSF for the same p‐tau assay. The strongest overall correspondence between plasma and CSF were observed for p‐tau217 assays (Figure 5), which had a rho range of 0.61–0.81 (all, *p*’s *<* 0.001) depending on the assay. However, when examining the AD pathology group alone, weaker and non‐significant associations where observed (rho = −0.042–0.36, *p* *>* 0.05; Figure 5). The only exception was the Fujirebio Lumipulse G p‐tau217 method, where CSF and plasma measures were significantly correlated in the AD group (rho = 0.4, *p* = 0.048). Similar findings were observed for p‐tau181 (Figure S6, Appendix p. 10), p‐tau231 (Figure S7, Appendix p. 11), and p‐tau212 (Figure S8, Appendix p. 12) with weaker and non‐significant correlations in the AD pathology group. Finally, we compared our plasma biomarker assays to the CSF p‐tau reference of this study (U.S. Food and Drug Administration (FDA)–approved Lumipulse G pTau181 in CSF (Figure S9, Appendix p. 13). In the whole group, 31 of 33 plasma assays correlated significantly with Lumipulse G CSF p‐tau181 (Figure S9A); however, in the AD pathology group alone, no assay significantly correlated with the reference CSF p‐tau181 biomarker (Figure S9B).

### 3.5 Candidate certified reference materials (CRMs)

A total of 25 plasma assays completed the measurement of the candidate CRM. Given the clear superiority of plasma p‐tau217 in the literature and this study, we report the commutability of the candidate CRMs for only those 10 p‐tau217 assays that completed CRM measurement (Figures S10 and S11, Appendix p. 14,15). In general, all candidate CRMs were not commutable (e.g., falling outside the 95% PI) for almost all method comparisons. No commutability was shown for p‐tau181 or p‐tau231 plasma assays (data not shown).

## 4 DISCUSSION

The specificity of plasma p‐tau to the pathologies underpinning AD^48^ offers great potential for its use as means of establishing a molecular diagnosis. It is important to note that an accurate and scalable tool for determining etiology will aid in improving clinical management by enhancing the differential diagnosis process and will reduce the need for more expensive and/or invasive PET scans or lumbar punctures. In this context, it is crucial to assess the performance of different p‐tau epitopes and compare different assays not only to each other but also to validated CSF markers of AD.

In this study, we performed a blinded comparison and commutability study of an unprecedented number (*n* = 33) of different plasma p‐tau measurements, including seven different p‐tau epitopes or p‐tau occupancy sites utilizing eight antibody‐based platforms. While the AUCs for all p‐tau217 assays were 0.94–1, another important metric for clinical use is the median fold‐change between two relevant groups (e.g., distinguishing AD from non‐AD pathology). Our findings clearly show that plasma p‐tau217, regardless of analytical method, had larger fold‐changes for determining the presence of AD pathology than p‐tau181, p‐tau205, p‐tau212, and p‐tau231. There was, however, variability in the median fold‐change across p‐tau217 assays. The UGOT IPMS p‐tau217, which simultaneously quantifies multiple p‐tau isoforms,^14^ Fujirebio Lumipulse G p‐tau217, a fully‐automated chemiluminescent method, and the Lilly MSD p‐tau217,^38^ a manual electrochemiluminescence method each provided a >5‐fold median increase in the AD pathology group. This was closely followed by the MSD S‐plex p‐tau217, ADx Simoa p‐tau217, Alamar NULISA p‐tau217, a nucleic acid linked immunoassay, and Roche Elecsys p‐tau217, a fully automated electrochemiluminescence method, which showed a median >4‐fold increase. The immunocapture diversity of these high‐performing methods demonstrates that the analytical method is not a critical factor in determining diagnostically accurate plasma p‐tau measures. In addition, for the first time, two fully automated random‐access immunoassays for p‐tau217 (Fujirebio Lumipulse G and Roche Elecsys), with shorter incubation times for higher analytical throughput, also produced top‐tier results in this study. Again, this points toward the importance of the composition and specificity of the assay design rather than the sensitivity of the ultra‐sensitive analytical platforms. This difference in fold‐increase between p‐tau217 tests is potentially important, given the Alzheimer's Association guidelines for blood biomarkers^49^ and the recent proposal for a two‐step workflow for the clinical implementation of blood biomarkers.^50^ This proposal acknowledges that a binary cutoff for AD plasma biomarkers would likely obtain sub‐optimal results and unacceptable numbers of false positives and false negatives.^51^ In a two‐step method, which identifies high‐risk and low‐risk individuals based on a risk model centered around p‐tau217, a biomarker with a larger fold‐change, will make interpretation easier and likely reduce numbers in an “intermediate” group, which would need confirmatory testing with CSF or PET imaging. An assay with a larger fold‐change will also be less susceptible to confounding factors^52^ impacting its diagnostic value.

We also used the same plasma biomarker assays to measure their CSF p‐tau counterparts. Previous studies have reported equivalence of diagnostic accuracy between CSF and plasma p‐tau assays.^18^, ^38^ Here, AUCs for AD pathology were also similar between plasma and CSF assays. Nevertheless, plasma p‐tau showed substantially lower fold‐changes for all biomarkers compared to their fold‐changes in CSF, which is expected given the proximity to the diseased organ. This stresses the need for considering aspects beyond AUC values when evaluating biomarker performance and diagnostic accuracy or when choosing a biomarker for local clinical implementation. In CSF, p‐tau217 and p‐tau212 showed larger fold‐changes compared to the other moieties. However, there was a narrower difference between plasma and CSF fold‐changes for p‐tau217 compared to those seen for p‐tau181, p‐tau212, and p‐tau231, suggesting that plasma p‐tau217 may more accurately reflect its CSF counterpart than other p‐tau markers, and thus, brain pathology.

The strong linear correlations between blood p‐tau217 assays, spanning multiple analytical platforms and assay designs, are notable and provide the potential to transition between assays, merge clinical data sets, and standardize the assays to each other using a CRM. However, in the AD pathology group alone, the associations were relatively poor, where correlations were non‐significant (except for one assay) and showed, at best, a Spearman's correlation coefficient of 0.4. Of note, it is important to bear in mind the limitations of a small sample size (*n* = 25 in the AD group). This was also observed when correlating all plasma biomarker assay measures to the reference standard CSF assay for p‐tau181 in this study. The apparent disconnect between plasma and CSF, for all assays, typically arose from higher‐than‐expected levels of plasma p‐tau in relation to the quantified CSF measurement and suggests that alternative mechanisms (e.g., blood–brain barrier impairment) may allow p‐tau to enter the bloodstream in an advanced disease stage that is independent of the amyloid and tau brain burden. Therefore, caution must be taken not to over interpret the meaning of absolute values of plasma p‐tau. Peripheral factors may also come into play in increased plasma p‐tau levels, and unexpectedly high plasma p‐tau values can also be observed in a single timepoint in healthy individuals followed over several weeks^23^ and in N‐terminal assay designs. Other more brain‐specific tau biomarkers such as brain‐derived tau (BD‐tau)^12^ or assays that are more reflective of tau pathology^13^, ^53^, ^54^ may provide further information in this context.

This study is not free of limitations. We fully acknowledge that the sample size is not sufficient to draw clear conclusions on diagnostic superiority (particularly among p‐tau217 assays), which was not the main aim of the study. We included only symptomatic individuals and did not select patients based on disease severity; and our participants did not undergo tau‐PET quantification, precluding us from examining whether some p‐tau biomarkers or assay designs are more associated with advancing disease severity or NFT pathology.^55^ Finally, the commutability aspect of the study was preliminary and designed prior to the inclusion of several different technologies in this rapidly developing field. Nonetheless, the lack of commutability of the candidate CRMs prepared here suggests that greater attention must be given to the details when developing and evaluating candidate CRMs, which will likely need to be phospho‐form‐specific. It is possible that differing calibration of the immunoassays and/or different measurands (i.e., the epitopes measured) may contribute to noncommutability between assays. The strong correlations between the different p‐tau217 assays suggest that further standardization work should focus on this marker, by testing a greater range of CRM formulations to optimize the CRM composition. Candidate CRMs will likely range from plasma pools spiked with CSF, to synthetic peptides for p‐tau217 sequences. Other possible enhancements to future efforts at standardization between assays would include the development of antibody‐free certified reference methods (e.g., as has been previously demonstrated to be feasible in CSF for p‐tau217 using a mass spectrometric technique^56^), which would eliminate cross‐reactivity between phosphorylation sites and variation in affinity across different antibody clones, and reduce matrix interference.

The Alzheimer's community can now call upon several plasma biomarker assays that can detect p‐tau forms in blood that strongly indicate the presence of AD pathology. This study, of the largest number of p‐tau assays to date, provides more evidence that assays targeting p‐tau217 using several different methodologies show good agreement with one another, and consistently demonstrate greater fold‐change in AD versus non‐AD groups than those targeting other p‐tau forms. These results show that this is not fundamentally predicated on a single analytical platform, or assay design. These findings confirm that plasma p‐tau217 may have clinical utility in determining the presence or absence of AD pathology in symptomatic individuals, which is relevant in the era of disease‐modifying therapies.

## CONFLICT OF INTEREST STATEMENT

All biomarker measurements were performed by the assay developers in‐house without cost. ALZpath p‐tau217 was performed at the University of Gothenburg (UGOT) and Lilly immunoassays were performed at the University of Lund. C_2_N Diagnostics declined to participate in the study. N.J.A. has given lectures in symposia sponsored by Eli‐Lilly, Roche Diagnostics, Alamar Biosciences, Biogen, VJDementia and Quanterix; consulted for Quanterix, TauRx, Neurogen Biomarking; served on advisory boards for Biogen, TauRx, and TargetALS; and has a pending patent application (PCT/US2024/037834 (WSGR Docket No. 58484‐709.601): Methods for Remote Blood Collection, Extraction and Analysis of Neuro Biomarkers). A.K., W.S.B., U.A., and B.A. have no conflicts of interest. M.D. is an employee of AbbVie and holds stock or stock options. S.B. is an employee of AbbVie and holds stock or stock options. J.V. is an employee of ADx NeuroSciences. C.L. is an employee of ADx NeuroSciences. M.V.L. is an employee of ADx NeuroSciences. E.S. is an employee of ADx NeuroSciences. S.I. is an employee of Alamar Biosciences. H.Y.J. is an employee of Alamar Biosciences. X.X. is an employee of Alamar Biosciences. A.F‐H. is an employee of Alamar Biosciences. B.Z. is an employee of, and has stock or stock options in, Alamar Biosciences. Y.L. is an employee of Alamar Biosciences. A.J. is former employee of ALZpath, Inc., and has stock in ALZpath, Inc., and Quanterix, Inc. M.V. is an employee of Fujirebio Europe N.V. N.L.B. is an employee of Fujirebio Europe N.V. H.K. is a former employee of Johnson and Johnson, and a current employee of the Enigma Biomedical Group; has undertaken paid consultancy for AviadoBio and Alector; has received an honorarium from Fortrea; and has a pending patient application for the Janssen CSF ptau217+ assay. D.B. has no conflicts of interest. G.T‐B. is an employee of Janssen R&D and has stock options; there is a pending patent application for the Janssen Simoa plasma p‐tau217+ assay. D.B. and S.J. have no conflicts of interest. S‐Y.Y. is an employee of MagQu Co., Ltd. C.D. is an employee of Meso Scale Diagnostics, LLC. D.R. is an employee of Meso Scale Diagnostics, LLC. G.S. is an employee of Meso Scale Diagnostics, LLC. J.W. is an employee of Meso Scale Diagnostics, LLC. K.M. is an employee of Quanterix. M.K. is an employee of Quanterix. A.J. is a full‐time employee of, and has stock or stock options in, Roche Diagnostics GmbH, Penzberg, Germany. L.S. is a full‐time employee of, and has stock or stock options in, Roche Diagnostics GmbH, Penzberg, Germany. J.G., P.R.K., F.G.‐O., and L.M.‐G. have no conflicts of interest. O.H. has acquired research support (for the institution) from C2N Diagnostics. In the past 2 years, he has received consultancy/speaker fees from AC Immune, BioArctic, Biogen, Bristol Meyer Squibb, Eisai, Eli Lilly, Fujirebio, Merck, Novartis, Novo Nordisk, Roche, Sanofi, and Siemens. R.A.R. has received consulting fees from Amydis Inc., Bioivt, Lexeo, Keystone Bio, Allyx, DiamiR, and PrecisionMed; and support for travel from Biogen. M.C.C. has no conflicts of interest. L.M.S. has served as a consultant or on advisory boards for Biogen, Roche Diagnostics, and Fujirebio; and receives in‐kind support from Fujirebio and Roche Diagnostics automated immunoassay platforms and reagents. K.B. has served as a consultant, on advisory boards, or on data monitoring committees for Abcam, Axon, BioArctic, Biogen, JOMDD/Shimadzu, Julius Clinical, Lilly, MagQu, Novartis, Ono Pharma, Pharmatrophix, Prothena, Roche Diagnostics, and Siemens Healthineers; and is a cofounder of Brain Biomarker Solutions in Gothenburg AB (BBS), which is a part of the GU Ventures Incubator Program, outside the work presented in this paper. J.M.S. has received research funding from Avid Radiopharmaceuticals (a wholly owned subsidiary of Eli Lilly); consulted for Roche Pharmaceuticals, Biogen, Merck, and Eli Lilly; given educational lectures sponsored by GE Healthcare, Eli Lilly, and Biogen; and is Chief Medical Officer for ARUK. H.Z. has served on scientific advisory boards and/or as a consultant for Abbvie, Acumen, Alector, Alzinova, ALZPath, Amylyx, Annexon, Apellis, Artery Therapeutics, AZTherapies, Cognito Therapeutics, CogRx, Denali, Eisai, Merry Life, Nervgen, Novo Nordisk, Optoceutics, Passage Bio, Pinteon Therapeutics, Prothena, Red Abbey Labs, reMYND, Roche, Samumed, Siemens Healthineers, Triplet Therapeutics, and Wave; has given lectures in symposia sponsored by Alzecure, Biogen, Cellectricon, Fujirebio, Lilly, and Roche; and is a co‐founder of Brain Biomarker Solutions in Gothenburg AB (BBS), which is a part of the GU Ventures Incubator Program (outside submitted work). COBAS and ELECSYS are trademarks of Roche. All other trademarks are the8 property of their respective owners. The Elecsys Phospho‐Tau (181P) CSF immunoassay is approved for clinical use. The Elecsys p‐Tau181 and p‐Tau217 prototype plasma immunoassays are not currently approved for clinical use or commercially available. Author disclosures are available in the Supporting Information.

## DATA AVAILAIBILITY STATEMENT

The anonymized data that support the findings of this study are available on request from qualified academic investigators, after approval of a proposal and with a signed data access agreement. Data will be shared for the sole purpose of replicating procedures and results. Requests should be directed to all three corresponding authors: a.keshavan@ucl.ac.uk, h.zetterberg@clinchem.gu.se and j.schott@ucl.ac.uk

## CONSENT STATEMENT

All human subjects provided written informed consent.

## Supporting information



Supporting Information

Supporting Information
